# Characterizing infection in anti-neutrophil cytoplasmic antibody–associated vasculitis: results from a longitudinal, matched-cohort data linkage study

**DOI:** 10.1093/rheumatology/keaa070

**Published:** 2020-03-11

**Authors:** Shifa H Sarica, Neeraj Dhaun, Jan Sznajd, John Harvie, John McLaren, Lucy McGeoch, Vinod Kumar, Nicole Amft, Lars Erwig, Angharad Marks, Corri Black, Neil Basu

**Affiliations:** k1 Aberdeen Centre for Health Data Science, University of Aberdeen, Aberdeen, UK; k2 Queen’s Medical Research Institute, University/British Heart Foundation Centre of Research Excellence, University of Edinburgh, Edinburgh, UK; k3 Department of Rheumatology, Raigmore Hospital, Inverness, UK; k4 Fife Rheumatic Diseases Unit, Whyteman’s Brae Hospital, Kirkcaldy, UK; k5 Centre for Rheumatic Diseases, Glasgow Royal Infirmary, Glasgow, UK; k6 Rheumatology Department, Ninewells Hospital, Dundee, UK; k7 GlaxoSmithKline, Medicines Research Centre, Stevenage, UK; k8 Morriston Hospital Renal Unit, Abertawe Bro Morgannwg University Health Board, Swansea, UK; k9 Institute of Infection, Immunity and Inflammation, University of Glasgow, Glasgow, UK

**Keywords:** granulomatosis with polyangiitis, eosinophilic granulomatosis with polyangiitis, microscopic polyangiitis, infections, longitudinal study

## Abstract

**Objectives:**

Infection exerts a major burden in ANCA-associated vasculitis (AAV), however, its precise extent and nature remains unclear. In this national study we aimed to longitudinally quantify, characterize and contextualize infection risk in AAV.

**Methods:**

We conducted a multicentre matched cohort study of AAV. Complementary data on infections were retrieved via data linkage with the population-based Scottish microbiological laboratory, hospitalization and primary care prescribing registries.

**Results:**

A total of 379 AAV patients and 1859 controls were followed up for a median of 3.5 years (interquartile range 1.9–5.7). During follow-up, the proportions of AAV patients with at least one laboratory-confirmed infection, severe infection and primary care antibiotic prescription were 55.4%, 35.6% and 74.6%, respectively. The risk of infection was higher in AAV than in matched controls {laboratory-confirmed infections: incidence rate ratio [IRR] 7.3 [95% confidence interval (CI) 5.6, 9.6]; severe infections: IRR 4.4 [95% CI 3.3, 5.7]; antibiotic prescriptions: IRR 2.2 [95% CI 1.9, 2.6]}. Temporal trend analysis showed that AAV patients remained at a higher risk of infections throughout the follow-up period, especially year 1. Although the *Escherichia* genus was the most commonly identified pathogen (16.6% of AAV, 5.5% of controls; *P* < 0.0001), AAV patients had the highest risk for *Herpes* [IRR 12.5 (95% CI 3.7, 42.6)] and *Candida* [IRR 11.4 (95% CI 2.4, 55.4)].

**Conclusion:**

AAV patients have up to seven times higher risk of infection than the general population and the overall risk remains significant after 8 years of follow-up. The testing of enhanced short- to medium-term prophylactic antibiotic regimes should be considered.

**Rheumatology key messages**

- AAV patients experience up to seven times greater risk of infection compared with matched controls.
- This risk is greatest in the first year but persists after 8 years of follow-up.
- The broad range of identified microbes indicates the potential need for broader-spectrum prophylactics.

## Introduction

ANCA-associated vasculitides (AAVs) are a group of multisystem autoimmune diseases comprising granulomatosis with polyangiitis, microscopic polyangiitis (MPA) and eosinophilic granulomatosis with polyangiitis. Until the adoption of immunosuppressive therapy in the 1980s, AAVs were almost always fatal; however, they are now considered chronic, relapsing diseases with estimated 5 year survival rates of 80% [1]. Patient prognosis in AAV remains poor—an observation that is often attributed to the consequences of drug toxicity, specifically infections [2–5].

The risk of infections in AAV is considered high; however, this has yet to be robustly quantified. Studies of infection in AAV report variable risks ranging from 6 to 67% [6–14]. This wide range is mainly ascribed to diverse sampling frames, with many studies focussing on uncontrolled, highly selected tertiary care populations. Moreover, different approaches to infection identification have been employed, with most limited to those resulting in hospitalization.

Currently there is no ideal method for assessing infection risk in a patient population. To attain a thorough understanding of the infection risk, it is essential to utilize complementary approaches involving different levels of healthcare, such as hospitalizations, microbiological laboratory records and antibiotic prescriptions, each measure bringing different methodological strengths and clinical insights. In Scotland, population-based healthcare records are routinely collected electronically and are centralized. Since the records of individuals are indexed under unique identification numbers, records in various healthcare registries can be linked. Capitalizing on this extensive data linkage capacity, in this large, matched-cohort study we aimed to contextualize infections in AAV by comparing them with those in the general population and, uniquely, to characterize infections in AAV in terms of severity, temporal trends and causative pathogens.

## Methods

### Routinely collected healthcare registries and data linkage in Scotland

National Health Services (NHS) Scotland holds rich population-based healthcare registries, with almost complete population coverage [15]. These centralized registries are routinely linked to follow up individuals within the healthcare system [16]. In this study we used the hospital episode data (SMR01), which records information on all day-case and inpatient hospitalizations in Scotland; the Electronic Communication of Surveillance in Scotland (ECOSS) registry, which collects information on all positive microbiological test results (from both primary and secondary care); and the Prescribing Information System database, which records all medications dispensed in primary care [17–20]. Data linkage was conducted by NHS Scotland via deterministic linkage methods using unique identification numbers. This process was previously demonstrated to be of high quality [21, 22].

### Governance

The research was approved by the Public Benefit and Privacy Panel for Health and Social Care, who scrutinize applications for access to anonymized routine data (1516-0194/Sarica). Information governance, confidentiality and data protection were undertaken according to the Data Protection Act (1998). Ethical approval was granted by the national Scotland ‘A’ Research Ethics Committee (15/SS/0152).

### Study design

We used a matched cohort study using routinely collected health data covering a 9 year period from 1 January 2008 to 28 February 2017. Approval was received from the Scotland ‘A’ Research Ethics Committee (reference 15-SS-0152) and the Public Benefit and Privacy Panel for Health and Social Care (reference 1516-0194).

### AAV cohort

An inception cohort of AAV patients was identified using the European Medicines Agency criteria in seven hospitals across Scotland [23]. To be eligible, patients were required to be diagnosed from 1 January 2008 onwards and be at least 16 years of age at diagnosis. Clinical information was collected on the date of diagnosis, including ANCA status and AAV type. The date of AAV diagnosis was assigned as the index date.

### General population controls

NHS Scotland matched each patient with AAV with up to five general population controls by age (±2 years), sex and geography, i.e. post code. Each patient in the general population cohort was assigned the same index date as their corresponding AAV patient.

### Follow-up period

Patients were followed from the index date until death or 28 February 2017, whichever came first. Information on death was collected through data linkage with the National Records System Death Registry, which records all deaths in Scotland [24].

### Identifying infections in routinely collected healthcare registries

Depending on the registry from which they were identified, infections were categorized as laboratory-confirmed infections, severe infections or primary care antibiotics prescriptions. Laboratory-confirmed infections were identified using free-text records in the ECOSS registry. Uniquely, this registry contains information on pathogens identified in microbiological specimens. Only information on the genus of each identified pathogen was feasibly extractable. We excluded pathogens identified using serological testing, as it was not possible to distinguish IgG positivity and thus reliably ascertain whether associated infective events actually occurred during follow-up. Severe infections were identified in the hospitalization database using the International Classification of Diseases, Tenth Revision algorithms developed by Inada-Kim *et al*. [25]. These algorithms were originally developed using administrative datasets for surveillance of patients with a risk of sepsis. Primary care antibiotics prescriptions were identified using relevant generic drug names and British National Formulary (BNF) paragraphs, the professional guidelines used for prescriptions in Scotland [26]. Details on relevant BNF paragraphs are available in Supplementary Table S1, available at *Rheumatology* online. To account for common prophylaxis against infections in AAV patients, we excluded azithromycin, co-trimoxazole and nystatin prescriptions in this analysis.

To minimize misclassification of prolonged infections as recurrent infections, we assumed that infection or antibiotics prescribing records within 28 days of each other represented a continued infection [27]. When ascertaining laboratory-confirmed infections, we incorporated data on causative pathogens as well. Accordingly, positive test results within 28 days of each other were considered recurrent infections if they were due to different pathogens. It was not possible to consider causative pathogens for severe infections and primary care antibiotic prescriptions, as relevant information was not available.

### Statistical analysis

The proportions of patients developing infections in each cohort were compared using chi-squared tests. Overall, infection rates in AAV and controls were compared using multilevel Poisson regression. The multilevel model introduces a ‘random effect’ that allows the risk of an event to vary randomly within each patient and hence permits repeated infections in the same patient to be independent [28, 29]. Models were adjusted for age at index, sex and health board of residence to account for confounding.

Temporal trends analysis was conducted by stratifying the follow-up time at 30, 90 and 180 days and yearly afterwards using Lexis expansions [30]. These time points were selected based on the current treatment guidelines on the duration of induction and remission therapy in AAV to provide sufficient granularity to observe potential temporal changes in the incidence rates of infections [23]. Infection rates at each interval were calculated separately by dividing the number of infections observed in each interval by person-years of follow-up included in each interval. The 95% CIs were computed using the Poisson assumption [28]. Incidence rate ratios (IRRs) comparing the rates at each discrete interval were calculated by dividing the rate in AAV by that in the general population. The 95% CIs around these IRRs were computed using the Byar method [31]. All analyses were performed in Stata version 14 (StataCorp, College Station, TX, USA) [32].

## Results

### Infections in AAV and general population controls

A total of 379 AAV cases were matched with 1859 general population controls. Patients were followed up for a median of 3.5 years. Baseline patient characteristics are shown in Table 1. The characteristics of patients included in the analysis pertaining to antibiotic prescription can be found in Supplementary Table S2, available at *Rheumatology* online.

**Table 1:** Patient characteristics at baseline

Characteristics	AAV (*n* = 379)	Controls (*n* = 1859)
Male sex, *n* (%)	196 (51.7)	965 (51.9)
Age at index, years, median (IQR)	61.6 (51.3–70.4)	61.5 (51.1–70.2)
Follow-up, years, median (IQR)	3.5 (1.9–5.7)	3.5 (2.0–5.7)
AAV type, *n* (%)		NA
GPA	205 (54.1)	
MPA	131 (34.6)	
EGPA	41 (10.8)	
Missing	2 (0.5)	
ANCA seropositivity, *n* (%)		NA
PR3-ANCA	185 (48.8)	
MPO-ANCA	149 (39.3)	
ANCA negative	43 (11.4)	
Missing	2 (0.5)	

EGPA: eosinophilic granulomatosis with polyangiitis; GPA: granulomatosis with polyangiitis; MPA; microscopic polyangiitis; PR-3: proteinase-3.

The proportions of AAV patients with at least one laboratory-confirmed infection, severe infection and primary care antibiotic prescription at follow-up were 55.4%, 35.6% and 74.6%, respectively (Table 2). Over the duration of the study, AAV patients were more likely than controls to develop laboratory-confirmed infections [adjusted IRR 7.3 (95% CI 5.6, 9.6)], develop severe infections [adjusted IRR 4.4 (95% CI 3.3, 5.7)] and receive primary care antibiotic prescriptions [adjusted IRR 2.2 (95% CI 1.9, 2.1)]. During follow-up, 35 cases died; none of these had infection listed as their main cause of death.

**Table 2:** Comparing overall infection incidence in AAV and the general population at follow-up

Factor	AAV, *n* (%)	Controls, *n* (%)	*P* for χ^2^ test	IRR (unadjusted) (95% CI)	*P* for IRR	IRR (adjusted)^a^ (95% CI)	*P* for IRR^a^
Laboratory-confirmed infections	210 (55.4)	294 (15.8)	<0.0001	6.7 (5.2, 8.6)	<0.0001	7.3 (5.6, 9.6)	<0.0001
Severe infections	131 (35.6)	202 (10.9)	<0.0001	3.8 (2.9, 5.1)	<0.0001	4.4 (3.3, 5.7)	<0.0001
Primary care antibiotic prescriptions^b^	258 (74.6)	902 (53.0)	<0.0001	2.1 (1.7, 2.5)	<0.0001	2.2 (1.9, 2.6)	<0.0001

General population controls were used as the reference group when calculating IRR.

a Adjusted for age, sex and health board of residence.

b Primary care prescribing data were available for 2009 onwards, therefore analysis included patients with an index date on or after 1 January 2009.

### Temporal trends of infections in AAV and general population controls

**Fig. 1:**
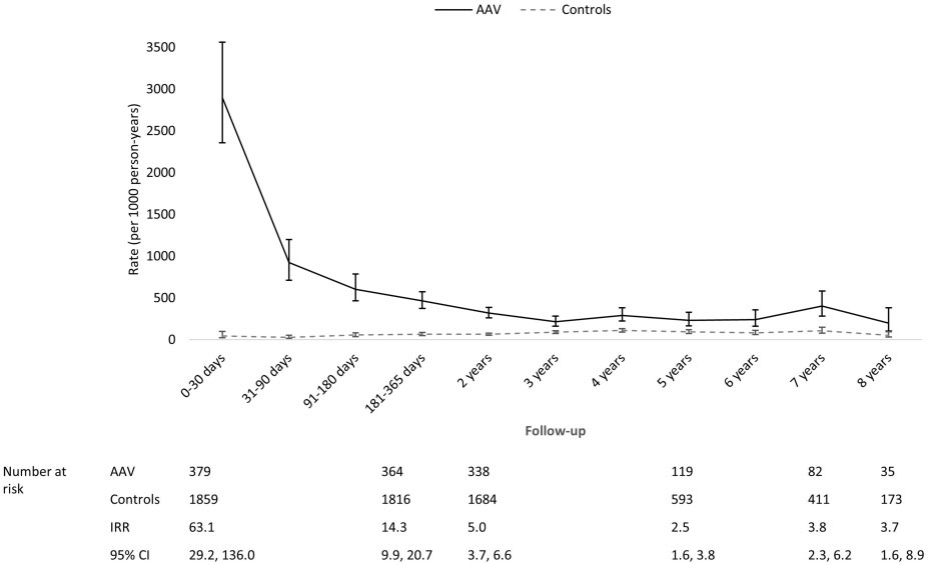
Comparing temporal trends in the rate of laboratory-confirmed infections in AAV and control The rate in controls was used as the reference when calculating IRR.

**Fig. 2:**
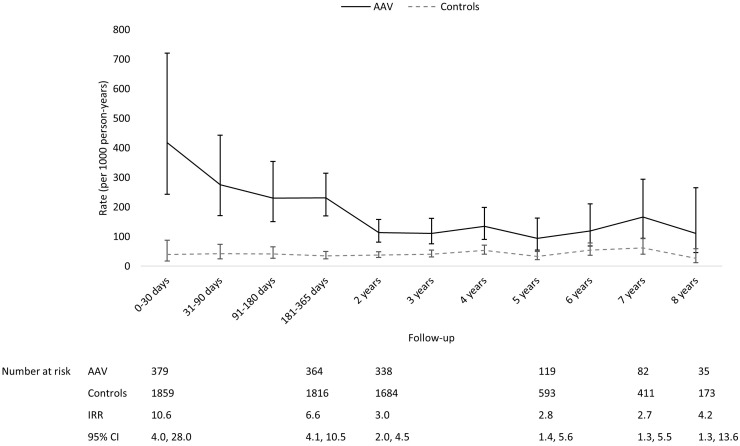
Comparing temporal trends in the rate of serious infections in AAV and controls The rate in controls was used as the reference when calculating IRR.

**Fig. 3:**
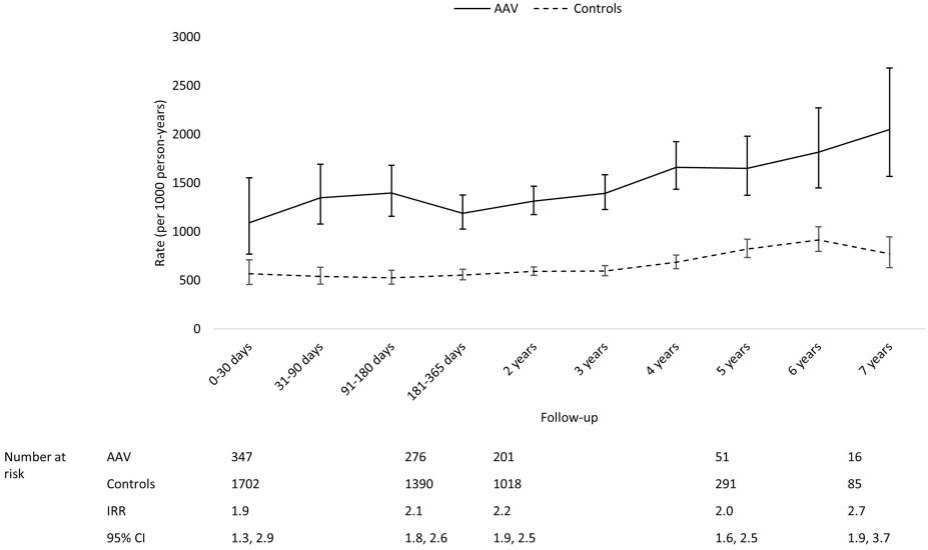
Comparing temporal trends in the rate of primary care antibiotic prescriptions in AAV and controls The rate in controls was used as the reference when calculating IRR.

AAV patients had the highest rates of laboratory-confirmed infections and severe infections during the first year of follow-up, especially during the first 30 days (Figs 1 and 2). Both the rates of laboratory-confirmed infections and severe infections fell thereafter; however, AAV patients remained at a higher risk of both types of infections than controls throughout follow-up. Although the difference in the rate of primary care antibiotics prescriptions between AAV and controls was not as substantial as the differences observed with the other metrics, throughout follow-up AAV patients had a higher rate of relevant prescriptions than controls (Fig. 3).

### Pathogens in AAV and the general population controls

Sixty-one pathogen types were identified during follow-up. AAV pathogens commonly involved the urinary tract (27%), followed by infections identified from the blood (22.3%) and lower respiratory tract (19.9%) (Supplementary Table S3, available at *Rheumatology* online). The risks of the most frequently observed pathogens in AAV are reported in Table 3. Results for rarer infections (*n* < 5) were not disclosed to preserve patient confidentiality; note that these included *Pneumocystis jirovecii*. Overall, *Escherichia* was the most commonly observed genus in both AAV and controls (16.2 *vs* 5.5%, *P* < 0.001). However, the greatest disparity in confirmed pathogens between AAV and controls were for *Herpes* [IRR 12.5 (95% CI 3.7, 42.6)], *Candida* [IRR 11.4 (95% CI 2.4, 55.4)], *Clostridium* [IRR 9.2 (95% CI 2.7, 30.7)], *Enterococcus* [IRR 5.3 (95% CI 2.2, 12.4)] and *Enterobacter* [IRR 5.2 (95% CI 1.0, 14.0)].

**Table 3:** Comparing pathogens in AAV and the general population

Infection type^a^	AAV (*n* = 379), *n* (%)	Control (*n* = 1859), *n* (%)	*P* for χ^2^ test	IRR (95% CI)	*P* for IRR	Adjusted IRR^c^ (95% CI)	*P* for IRR^c^
*Escherichia*	63 (16.6)	102 (5.5)	<0.0001	4.9 (3.2, 7.5)	<0.0001	4.3 (2.7, 6.9)	<0.0001
*Staphylococcus*	49 (12.9)	64 (3.4)	<0.0001	5.1 (3.1, 8.5)	<0.0001	5.0 (3.3, 7.5)	<0.0001
*Klebsiella*	26 (6.9	34 (1.8)	<0.0001	4.9 (1.7, 14.1)	0.003	4.7 (2.1, 10.6)	<0.0001
*Haemophilus*	23 (6.1)	30 (1.6)	<0.0001	4.3 (2.3, 8.1)	<0.0001	5.7 (3.3, 10.0)	<0.0001
*Streptococcus*	20 (5.3)	29 (1.6)	<0.0001	4.8 (2.7, 8.8)	<0.0001	5.5 (3.2 ,9.3)	<0.0001
*Enterococcus*	19 (5.0)	41 (2.2)	0.002	5.3 (2.2, 12.4)	<0.0001	6.8 (2.9, 16.0)	<0.0001
*Candida*	9 (2.4)	12 (0.7)	0.001	11.4 (2.4, 55.4)	0.002	NA	NA
*Enterobacter*	8 (2.1)	7 (0.4)	<0.0001	5.2 (2.0, 14.0)	0.001	NA	NA
*Herpes* ^b^	7 (1.9)	<5 (<0.3)*	<0.0001	12.5 (3.7, 42.6)	<0.0001	NA	NA
*Pseudomonas*	7 (1.9)	7 (0.4)	0.001	2.0 (0.5, 7.6)	0.326	NA	NA
*Citrobacter*	6 (1.6)	<5 (<0.3)*	<0.0001	4.8 (1.4, 16.7)	0.014	NA	NA
*Clostridium*	6 (1.6)	<5 (<0.3)*	<0.0001	9.2 (2.7, 30.7)	<0.0001	NA	NA
*Campylobacter*	5 (1.3)	7 (0.4)	0.022	3.4 (1.6, 7.2)	0.002	NA	NA
*Mycobacterium*	5 (1.3)	6 (0.3)	0.011	3.8 (0.8, 17.8)	0.091	NA	NA

a Infection types were determined using genus only. Therefore it is not possible to distinguish between different types of infections of the same genus, e.g. herpes simplex virus infections *vs* herpes zoster virus infections.

b Includes all herpes viruses.

c Adjusted for age at index, sex and health board of residence. Infection types are ordered from most to least common infections in AAV.

* Cell values <5 suppressed in accordance with statistical disclosure process to preserve patient confidentiality.

NA: model adjustment was not applicable due to small numbers.

## Discussion

In this study we are the first to longitudinally quantify and characterize the infection risk in AAV and compare it with that in the general population using three complementary infection measures. Our findings show that AAV patients are at a high risk of infections: they were seven times more likely than the general populations to have laboratory-confirmed events, four times more likely to develop infections requiring hospitalization and twice as likely to have been prescribed antibiotics in primary care. Crucially, our data also indicate that this increased risk persists in the long term. While *Escherichia* was the most common pathogen identified in both AAV and controls, the greatest proportional differences between the two groups were seen for *Herpes*, *Candida*, *Clostridium*, *Enterococcus* and *Enterobacter*.

Our findings are consistent with previous studies that have largely focussed on severe infections requiring hospitalization. For instance, a Swedish study showed an increased risk of severe infections in AAV compared with the general population using similar methods [11].

Our long-term assessment of infection risk in AAV is novel. While several studies agree that the risk of infection in AAV is greatest early after diagnosis when the burden of immunosuppression is greatest [33], we are the first to demonstrate that this risk remains elevated for several years. It is not uncommon for patients to remain on immunosuppression for this length of time. However, a large proportion of patients are completely weaned off such therapy after 2–5 years and it is interesting to observe similar infection risk ratios from year 3 onwards, suggesting persisting long-term immune dysfunction that may reflect chronic perturbation of the underlying disease or continued injury from past immunosuppression (although it is noteworthy that during the time frame of this study, rituximab was not commonly prescribed in Scotland).

Controlled study designs are crucial in infection epidemiology, and here we report the first comparisons of pathogens between AAV and the general population. We show that bacterial infections are the most commonly observed infections in AAV. Although limited in number, previous uncontrolled single-centre studies have indicated the same [7, 12, 13, 34, 35]. The largest of these studies was by Yang *et al.* [12], who reviewed the case notes of 248 AAV patients in China. Of the 87 infections observed within a 15 month period, *Acinetobacter baumannii*, *Staphylococcus aureus* and *Pseudomonas aeruginosa* constituted the most common bacterial pathogens, while *Candida albicans* and varicella-zoster virus were the most common fungal and viral pathogens, respectively. In our study, the most common bacterial pathogens were *Escherichia*, *Staphylococcus* and *Klebsiella*, while the most common viral and fungal pathogens were *Herpes* and *Candida*. Our study excluded episodes of varicella-zoster and many other viruses detected by Ig testing, as it was not possible to distinguish IgG from IgM responses. The differences in bacterial pathogens between the two studies may be due to differences in geography and follow-up times (a median of 3.2 years in our study *vs* 15 months).

Our study has several important strengths. First, we used three different methods, each with its advantages, and identified infections in rich healthcare databases with almost complete population coverage. In particular, the largely consistent temporal trends of infection risk across laboratory-confirmed and severe infections provide validity to our conclusions. We did not observe a similar disparity between the earlier rates of primary care antibiotics prescribing in AAV and the general population, an observation that most likely reflects the predominance of hospital-directed management during the initial post-diagnosis phase.

Several methodological limitations must be considered when interpreting our findings. Our study was not entirely population-based. However, we identified AAV patients from rheumatology and nephrology departments, the two departments primarily responsible for managing AAV patients in Scotland, and the sites involved in our study included teaching and general hospitals from almost every health catchment area in Scotland, thus the potential impact of selection bias on our findings is likely minimal. Second, the effect of potential surveillance bias on the results must also be considered. Infection among immunosuppressed patients is a common concern and so clinicians will have a lower threshold to investigate and treat AAV patients for this possibility compared with the general population. This explains some of the contextual disparity with the general population in regards to the laboratory-confirmed infections and antibiotic prescriptions, although such bias will be a less of an issue for serious infections. Finally, while data linkage of administrative databases offers an efficient and cost-effective way to develop and analyse disease cohorts, researchers with specific hypotheses are inevitably constrained to the analysis of variables originally determined for alternative purposes. For this reason, we were unable to report potentially interesting data such as numbers of hospital-acquired infections, immunosuppressant burden, prevalence of renal dysfunction and vasculitis relapse.

Even in the absence of quantitative data, physicians have long been familiar with the risk of infections in AAV. Indeed, international recommendations for the management of vasculitis advocate prophylaxis against infections (specifically co-trimoxazole for *P. jirovecii*, which was prescribed to 76% of this cohort) in patients taking cyclophosphamide and routine checks for hypoimmunoglobulinaemia prior to each course of rituximab, as well as vaccination against certain infections, if possible [33, 36]. Despite these efforts to tackle the infectious burden in AAV, the current study demonstrates that this risk remains substantial. It is highest immediately following diagnosis and the spectrum of pathogens at this time is broad. Moreover, the risk remains significantly greater than in the general population, even after 8 years. Taken together, the role of broader-spectrum antibiotic prophylaxis—at least during the first months—should be considered. For example, low-dose cephalexin has been safely and effectively used for infection prophylaxis in other populations [37] and its spectrum of action is known to target the salient microbes identified herewith. The gains of such an intervention must be balanced with their toxicity (e.g. higher rates of *Clostridium*) and so clinical trials are warranted to complement targeting of modifiable risk factors (e.g. corticosteroids, dosing regimens) in order to reduce this unacceptable infection burden.

## Supplementary Material

keaa070_Supplementary_Data
