# Supplementary material for: Characterizing infection in anti-neutrophil cytoplasmic antibody–associated vasculitis: results from a longitudinal, matched-cohort data linkage study
Source: Rheumatology (Oxford). 2020 Mar 11;59(10):3014–22. doi: 10.1093/rheumatology/keaa070 (PMC7516107; doi:10.1093/rheumatology/keaa070)
Supplement: keaa070_Supplementary_Data [file keaa070_supplementary_data.docx]

**SUPPLEMENTARY MATERIAL**

**Supplementary Table S1. British National Formulary Paragraphs used for identifying antibiotic prescriptions**

| **Category** | **BNF paragraph** |
| --- | --- |
| Penicillins | 5.1.1 |
| Macrolides | 5.1.5 |
| Cephalosporins | 5.1.2.1 |
| Aminoglycosides | 5.1.4 |
| Tetracyclines | 5.1.3 |
| Quinolones | 5.1.12 |
| Antifungals | 5.2 |
| Anti-tuberculosis drugs | 5.1.9 |
| Other: | |
| Trimethoprim | 5.1.8 |
| Metronidazole | 5.1.11 |
| Chloramphenicol | 5.1.7 |
| Aciclovir | 13.10.3 |

The following prescriptions were excluded: azithromycin, co-trimoxazole, and nystatin.

**Supplementary Table S2. Characteristics of the 2009 cohort**

| Characteristic | AAV  (n=347) | Controls  (n=1702) |
| --- | --- | --- |
| Male sex, n (%) | 182 (52.5) | 895 (52.6) |
| Age at index, years (median; IQR) | 62.0 (51.5, 70.4) | 62.0 (51.8, 70.6) |
| AAV TYPE, n (%)  GPA  MPA  EGPA  Missing | 187 (53.9)  123 (35.4)  36 (10.4)  1 (0.3) | NA |
| ANCA SEROPOSITIVITY, n (%)  PR3-ANCA  MPO-ANCA  ANCA negative  Missing | 169 (48.7)  140 (40.4)  37 (10.7)  1 (0.3) | NA |

AAV: ANCA-Associated vasculitis; ANCA: Anti-neutrophil cytoplasmic antibody; EGPA: Eosinophilic Granulomatosis with Polyangiitis; IQR: Interquartile range; GPA: Granulomatosis with polyangiitis; MPA; Microscopic polyangiitis; PR-3: Proteinase-3; MPO: Myeloperoxidase.

**Supplementary Table S3. Distribution of specimen site for laboratory-confirmed infections in 210 AAV patients**

| **Specimen site** | **n (%)** |
| --- | --- |
| Blood | 147 (23.9) |
| Integumentary | 26 (4.2) |
| Upper respiratory tract | 35 (5.7) |
| Lower respiratory tract | 147 (23.9) |
| Gastrointestinal tract | 33 (5.4) |
| Urinary Tract | 208 (33.9) |
| Not specified | 18 (2.9) |

AAV: ANCA-associated vasculitis.

**Supplementary Table S4 Distribution of specimen site for laboratory-confirmed infections in AAV patients**

| Specimen site | n (%) |
| --- | --- |
| Blood | 147 (23.9) |
| Integumentary system | 26 (4.2) |
| Upper respiratory tract | 35 (5.7) |
| Lower respiratory tract | 147 (23.9) |
| Gastrointestinal tract | 33 (5.4) |
| Urinary Tract | 208 (33.9) |
| Not specified | 18 (2.9) |

AAV: ANCA-associated vasculitis.
